# A Comparative 3D Finite Element Computational Study of Three Connections

**DOI:** 10.3390/ma12193135

**Published:** 2019-09-26

**Authors:** Davide Farronato, Mattia Manfredini, Andrea Stevanello, Veronica Campana, Lorenzo Azzi, Marco Farronato

**Affiliations:** 1 School of Dentistry, Department of Medicine and Surgery, University of Insubria, 21100 Varese, Italy; davide@farronato.it (D.F.); veronica.campana93@gmail.com (V.C.); 2 Corso Europa 10, 20122 Milan, Italy; mattiamanfredinidr@gmail.com; 3 Department of Medicine and Surgery, University of Insubria, Unit of Oral Pathology, Dental Clinic, ASST dei Sette Laghi, 21100 Varese, Italy; lorenzoazzi86@hotmail.com; 4 IRCCS Fondazione Cà Granda, University of Milan, Via francesco Sforza 28, 20122 Milan, Italy; marcofarronato@msn.com

**Keywords:** connection, implant–abutment interface, FEM analysis, stress distribution

## Abstract

Masticatory overload on dental implants is one of the causes of marginal bone resorption. The implant–abutment connection (IAC) design plays a critical role in the quality of the stress distribution, and, over the years, different designs were proposed. This study aimed to assess the mechanical behavior of three different types of IAC using a finite element model (FEM) analysis. Three types of two-piece implants were designed: two internal conical connection designs (models A and B) and one internal flat-to-flat connection design (model C). This three-dimensional analysis evaluated the response to static forces on the three models. The strain map, stress analysis, and safety factor were assessed by means of the FEM examination. The FEM analysis indicated that forces are transmitted on the abutment and implant’s neck in model B. In models A and C, forces were distributed along the internal screw, abutment areas, and implant’s neck. The stress distribution in model B showed a more homogeneous pattern, such that the peak forces were reduced. The conical shape of the head of the internal screw in model B seems to have a keystone role in transferring the forces at the surrounding structures. Further experiments should be carried out in order to confirm the present suppositions.

## 1. Introduction

Dental implantology is a well-established treatment option in cases of missing teeth, showing good results in terms of long-term success rates [1]. However, failures are still encountered in early and late time-points [2]. Several causes were addressed for implant failure and the implant biomechanical behavior is considered one of the determining factors affecting the implant longevity [3]. Masticatory overloads on an implant were pointed out as a possible cause of marginal bone resorption, due to the excessive stresses generated in the peri-implant tissue [4]. The transfer and the distribution of biomechanical loads are highly affected by the design of the constituting components and materials [5,6,7,8]. The implant–abutment connection (IAC) plays a critical role in the quality of the stress distribution, and, over the years, different designs were proposed in order to control the stress distribution [6,9].

Implants have a rigid and tight interface because of the absence of a periodontal ligament; thus, loading applied to the implant system is directly transferred to the bone [10,11]. The reduction of stresses at the IAC interface may prevent biomechanics features of the rehabilitation, such as component fracture, screw loosening, and augmented leaking at the connection interfaces [12].

Finite element method (FEM) analysis is a common procedure in order to study the mechanical behavior of dental implants, especially the stress distribution generated at the IAC interface. Previous studies observed how a particular design may affect the stress distribution at the connection interface [12,13,14,15,16,17,18,19].

For this reason, three different types of IAC were compared in an FEM analysis. The aim of this study was to determine how the IAC design may affect the stress distribution among different implant components and peri-implant tissues.

## 2. Materials and Methods

**Figure 1:**
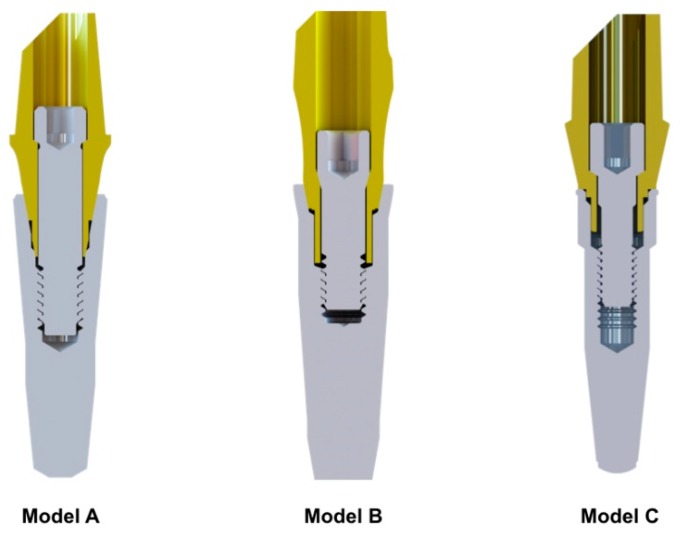
The three types of implant connections evaluated: two internal 11° conical connections (models A and B) and one internal flat-to-flat connection implant (model C).

Three types of implants were considered: two internal 11° conical connections (model A and model B) and one internal flat-to-flat connection implant (model C) (Figure 1). The outline shape of all samples was in root form.

All of the implants compared in this study were chosen with similar diameters at the implant neck such to reduce the bias. The diameters of models A, B, and C were respectively 4 mm, 4.2 mm, and 3.8 mm. Moreover, the position of the screw head inside the abutment and the angle of contact between the screw and the abutment were different between model A and model B. The angles were respectively 59° and 15°; the contact between the screw and abutment in model A was designed more distant from the connection, and the screw was consequently higher.

The average thickness of the connection stress zone was equal to 0.55 mm for all models.

**Figure 2:**
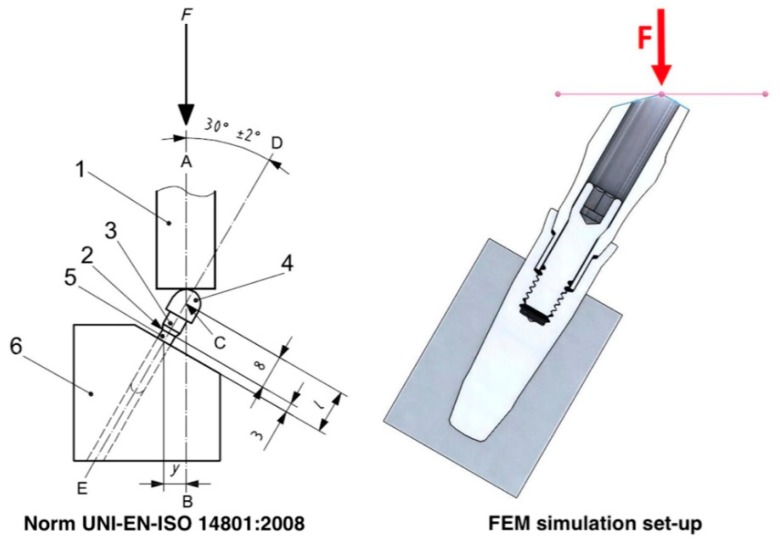
Loading geometry in accordance with standard UNI-EN-ISO 14801:2008 (1: loading device, 2: nominal bone level, 3: abutment, 4: hemispherical loading member, 5: dental implant body, 6: specimen holder, F: loading force, C: loading center, AB: loading axis, DE: dental implant axis). In the FEM simulation set-up, the loading geometry was modeled with a point load in the position shown on the right.

The loading geometry simulated through the FEM models was chosen in order to emulate the experimental set-up prescribed by the standard UNI-EN-ISO 14801:2008 (Figure 2). The examined samples presented no threads so as to exclude the latter’s influence when comparing the connection’s biomechanics.

Each model was composed of a cylindrical holder, where the implant was inserted and connected with a straight abutment through the internal screw. The material compositions of the implant, abutment, screw, and holder were respectively titanium grade 4, titanium grade 5, titanium grade 5, and 1060 aluminum alloy. For the holder, aluminum alloy was chosen in order to have a Young’s modulus lower than that of titanium, the material used for the implant–abutment system. Models A and B are prototype projects (Prodent Italia S.r.l, Pero, Milan, Italy), and model C is a commercialized implant (Prime, Prodent Italia S.r.l Pero).

As requested by the standard, the holder should be chosen in order to ensure that no permanent deformation occurs, but this material does not simulate the bone. The FEM comparisons performed on different types of connections are meant to be static and qualitative with the purpose of forecasting different possible mechanical characteristics.

The geometry of all components was shaped with SolidWorks 2016 (SolidWorks Corp, Waltham, MA, USA). For simplification reasons, the threaded parts of the system were neglected, and the implants were shaped considering the dimension of the pitch diameter.

This may be a valid simplification because this study does not focus on the interaction of the implants with bone, but just on the mechanical behavior of the IAC. Furthermore, the threaded part makes the FEM calculation more complex without having additional value; thus, it was omitted in all the models, neglecting its influence for the comparison between implant geometries.

In accordance with the standard, in the coronal portion of the abutments, a 30° cut was present, referred to the main axis of the implant, so as to introduce a 30° tilting force when an axial force was applied. The force was applied at a distance of 11 mm from the connection (Figure 2).

The geometries were imported into the finite element software SolidWorks Simulation (SolidWorks Corp, Waltham, MA, USA) in order to generate the meshes with tetrahedral solid elements. The total numbers of nodes and elements of each model are described in Table 1; the mechanical properties of the implant, abutment, screw, and holder are summarized in Table 2. All materials were considered isotropic and linearly elastic. Further specific material characterizations were set according to the software libraries.

No-penetration contacts were set between the surfaces of the screw and implant–abutment, the surfaces of the implant and abutment, and among implant–holder components. Symmetry boundary conditions were used, because of the mirror symmetry of the geometry of interest. A compression load of 175 N was applied to the surface of the abutment.

The following qualitative analyses were performed in the FEM: deformation maps, von Mises stress maps, and safety factor maps. These tests were not suitable for quantitative measurements, due to the simplifications considered during the setting phase of the analysis.

## 3. Results

### 3.1. Strain Map

**Figure 3:**
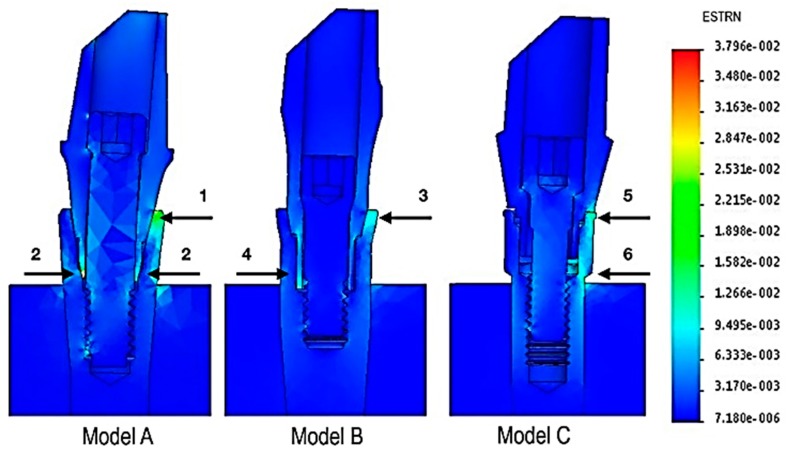
Strain map: the arrows indicate the areas of major strain, whose values are expressed by a gradient color scale.

The FEM analysis (Figure 3) showed that model A presented two critical points where the local values of strain were highest. These points were the fulcrum zone located at the coronal junction between the abutment and implant, in the area corresponding to the direction of the driving force vector (arrow 1), and the area opposed to the direction of this force was represented by the connection part of the abutment (arrow 2). In model B, the lowest resistance appeared at two points, both corresponding to the connection surfaces: one located at the coronal level (arrow 3) and one at the apical level (arrow 4). The position of the weakest points seemed to be similar to model A, with a lower strain value.

In model C, the weakest points were the fulcrum area, which was located at the most coronal contact point between abutment and implant (arrow 5), and the whole area in the direction of the driving force vector (arrow 6).

### 3.2. Stress Analysis

**Figure 4:**
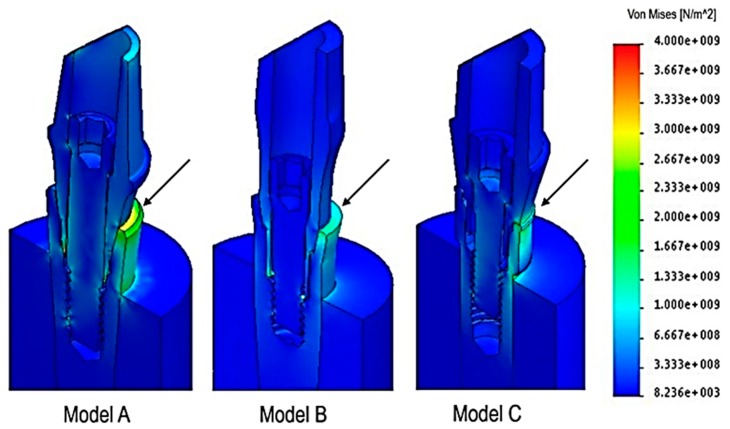
Stress map: the arrows indicate the areas of major strain, whose values are expressed by a gradient color von Mises scale.

The von Mises stress (Figure 4) indicated how the load was distributed on the different surfaces and, consequently, which areas were more susceptible to stress. Load was mostly distributed on the implant connection area for all three models (arrows shown in Figure 4). However, it can be seen that model B appeared to achieve lower von Mises values than the other two models, as well as a reliable connection. The forces were more distributed on a wider surface, and the internal screw seemed less affected by mechanical stresses.

### 3.3. Safety Factor

**Figure 5:**
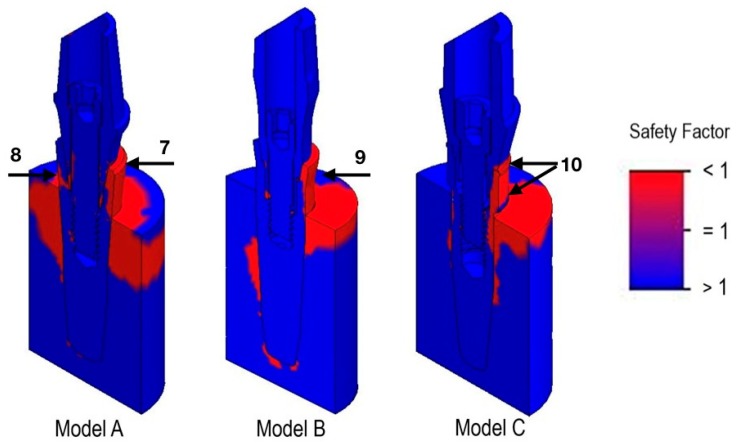
Safety factor map: the most critical zones are indicated in red.

The safety factor (SF) map is shown in Figure 5.

In model A, the most critical areas appeared to be the most coronal part of the implant–abutment interface according to the force vector appliance (arrow 7) and the most apical site of the connection in the contralateral zone at the neck of the implant (arrow 8). In model B, the most critical parts were represented by the neck of the implant (arrow 9) and in the most apical portion of the connection in the contralateral side. In model C, the most dangerous zones were at the implant neck and at the coronal part of the body (arrow 10).

### 3.4. Implant Model Comparison

Model A showed less dissipation of forces, resulting in a decrease in total resistance. In addition, the internal screw acted as a retentive element counteracting the force. The internal screw, which represented an effective but isolated point of resistance, seemed to be hollow, further reducing stress distribution.

The model A design has a power arm (distance between fulcrum and driving force) longer than the resistance arm (distance between fulcrum and resistance), which turned out to be an advantageous lever, avoiding internal screw potential problems. Furthermore, the stabilization cone positioned under the anti-rotational element seemed to not be thick enough in order to manage the loads at an angled vector application.

In model B, the distribution of stress seemed to show no overload peaks with no particularly risky areas.

The lever arm, although shorter than that in model A (power arm), was greater than the resistance arm, and it resulted in a favorable configuration.

In model C, the abutment–implant connection was guaranteed by the internal screw, as this connection was not provided with an intrinsic retention. Thus, any tilting force and micromotion seemed to affect the resistance and mechanical stability of this internal screw.

This last connection showed a tendency to transmit loads and deformations in depth and along the body of the internal screw, as well as having a risk of overloading concentrated in a single area.

## 4. Discussion

From the present qualitative and comparative FEM analysis, it could be observed that the model B connection seemed to show better mechanical behavior compared to the conical connection of model A and the internal flat-to-flat connection of model C.

The FEM analysis indicated that most of the forces were transmitted on the abutment and implant’s neck in model B, whereas, in model A and model C, forces were distributed along the internal screw, abutment areas, and implant’s neck.

Stress distribution at the level of the implant abutment connection is strongly associated with the design characteristics of the interface [14,20]. The chosen models presented minimal diameter differences at their widest point, which may have partially affected the results [21]. Moreover, it may be considered that the outline of different implant projects differed not only in the widest measurable diameter, but also in the average thickness of the connection stress zone. Due to this reason, the three models were evaluated to be compatible to a comparison because the average thickness of the connection stress zone, belonging to all the three, was equivalent, despite the wider diameter.

The advantages and disadvantages of the different connection types were studied by several authors [22,23,24,25].

Coppede et al. demonstrated that the friction-locking mechanics of the internal conical connections provided greater resistance to deformation and fracture under oblique compressive loading when compared to the internal hexagon connections [23].

Balik et al. showed that a screw tapered connection compared with an internal connection, external connection, and cone Morse connection revealed the lowest strain values. These previous reports are consistent with the present FEM analysis study where the conical hex connection of model B showed the lowest strain values [14].

Norton et al. reported that internal conical connections increased resistance to bending moments at the fixture–abutment interface when compared to a butt joint interface [25].

Moreover, the micro gap between the implant and the abutment causes an increase of stress distribution on the connection components, implant, and surrounding bone [26,27,28]. The gap between the implant and abutment interface depends on the connection’s design [29,30]. This affects the implant connection from both a biological and mechanical point of view.

The biological problem is related to potential risk for invasion of oral microorganisms into the fixture–abutment microgap, allowing bacteria to penetrate and colonize the inner part of the implant, and this fact, in vivo, produces a bacterial reservoir that could interfere with the long-term health of the peri-implant tissues [31,32,33].

The mechanical problem of the microgap is related to a possible loosening or fracture of the internal screw caused by micro movements. Several studies [23,34] demonstrated that the internal conical implant–abutment connection is mechanically more stable than a flat-to-flat one, and able to provide a better seal [35].

The present FEM analysis showed that the seal quality of model C, for the deformations shown during force loading, seemed reduced, even if the present study represents a qualitative assessment rather than a quantitative measure. According to the present study and the literature, the relative risks that could develop on this kind of connection are represented by the unscrewing of the internal screw or its breaking, the fracture of the implant, and the bacterial infiltration [32,36].

Scarano et al. observed that, in the area of the internal conical connection implants, there was no detectable separation at the implant/abutment, and no presence of a microgap [37]. Farronato et al. observed the same for a static angled force application [38]. However, in the internal hexagon connection, numerous voids were present between the implant and abutment interface.

In an in vitro study, Scarano et al. observed that conical Morse taper connections were shown to be tighter and more stable from a biomechanical point of view than flat-to-flat connections [39].

According to these studies, model B’s conical connection showed better distribution of the force along the whole connection caused by high contact between the abutment and internal surface of the implant. Although model A had an internal conical connection, the strain map (Figure 3) seemed to show a lower seal quality than in model B.

On the other hand, Coelho et al. demonstrated that micro-leakage at the implant/abutment interface was shown to occur in all implant systems with variability between the different systems. The presence of a microgap could be due to an incorrect machining of the component parts and to excessive torque forces during the insertion of the fixture [27].

The FEM analysis of the present study showed that loading forces could affect the internal screw. The strain map (Figure 3) reported deformation of the internal screw in model A and model C, compatible with traction and bending. The conical internal screw of model B seemed not to be involved during loading forces, avoiding risks of screw overloading or loosening. Coppede et al. observed in an in vitro study that the use of conical-head abutment screws with frictional locking action resulted in greater connection stability and mechanical resistance compared to conventional flat-head screws, regardless of the connection design [40].

**Figure 6:**
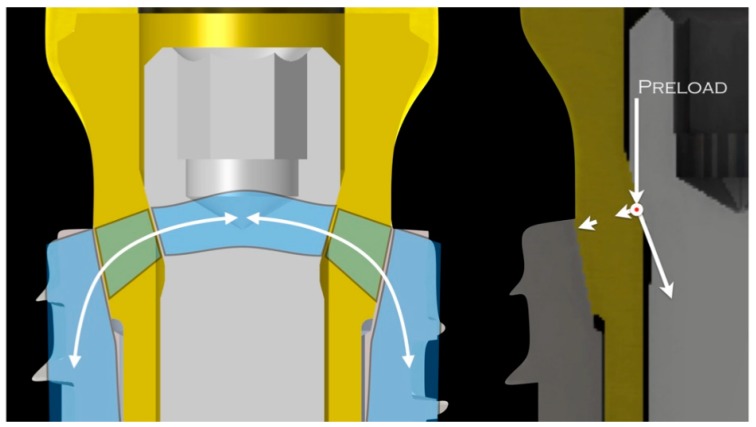
Model B connection: the internal screw has a role akin to a keystone in an arch system. It transfers the forces at the surrounding structures without interposed gaps.

The model B connection has a design comparable to a keystone arch (Figure 6).

This system, known for millennia and used in buildings to dissipate the forces, is the result of the union between implant, abutment, and conical internal screw. A different inclination degree between implant and abutment (11°) and between abutment and internal screw (15°) seems to represent a key factor for the distribution of forces in a homogeneous way. Specifically, the shape of the head of the internal screw seems to have a role akin to a keystone in an arch so as to transfer forces at the surrounding structures without interposed gaps. It is assumed that this design can subdivide the forces between the various components of the connection in such a way that the resultant of the forces does not develop and concentrate in a single point but at different points, reducing the risk of overloading. Moreover, during mechanical loads, model B acts as a single body, reducing possible tilting forces that could create a gap between the structures of the connection, which is why it differs from the taper-locking model A. According to the literature, a conical-head internal screw prevents microbial leakage through the implant–abutment interface, ensuring a better seal [41]. One of the limitations of this study is that we focused exclusively on implant connections and, for this reason, the threaded parts of the system were neglected. In addition, further investigations may be encouraged so as to evaluate the effect of different combinations of angles between the implant and abutment and between the abutment and internal screw, in order to detect the most efficient load distribution.

## 5. Conclusions

Although characterized by mechanical and computational limits, this analysis seems to show the potential advantages of the model B connection from a biomechanical point of view. The distribution of stress is more homogeneously shared, and the force peaks are reduced, making it theoretically safer. Further experimental load tests should be carried out in order to confirm the present suppositions. The importance of FEM computational studies, such as the present one, in the domain of dental implantology is to qualitatively study the behavior of different geometrical and dimensional configurations of implants and implant–prosthetic connections, with the aim to select those to be subjected to experimental tests.

## Figures and Tables

**Table 1:** Nodes and elements.

Model	Nodes	Elements
A	432,329	296,377
B	253,773	164,806
C	173,404	111,916

**Table 2:** Materials and mechanical properties.

Structures	Material	Young’s Modulus (GPa)	Poisson’s Ratio
Implant	Titanium grade 4	105	0.37
Abutment	Titanium grade 5	110	0.3
Screw	Titanium grade 5	110	0.3
Holder	1060 aluminum alloy	69	0.33
